# Technical Note: Using k‐means clustering to determine the number and position of isocenters in MLC‐based multiple target intracranial radiosurgery

**DOI:** 10.1002/acm2.12139

**Published:** 2017-07-20

**Authors:** Adam D. Yock, Gwe‐Ya Kim

**Affiliations:** ^1^ Department of Radiation Oncology Vanderbilt University Medical Center Nashville TN USA; ^2^ Department of Radiation Medicine and Applied Sciences University of California San Diego La Jolla CA USA

**Keywords:** machine learning, multiple target, single isocenter, stereotactic radiosurgery

## Abstract

**Purpose:**

To present the k‐means clustering algorithm as a tool to address treatment planning considerations characteristic of stereotactic radiosurgery using a single isocenter for multiple targets.

**Methods:**

For 30 patients treated with stereotactic radiosurgery for multiple brain metastases, the geometric centroids and radii of each met were determined from the treatment planning system. In‐house software used this as well as weighted and unweighted versions of the k‐means clustering algorithm to group the targets to be treated with a single isocenter, and to position each isocenter. The algorithm results were evaluated using within‐cluster sum of squares as well as a minimum target coverage metric that considered the effect of target size. Both versions of the algorithm were applied to an example patient to demonstrate the prospective determination of the appropriate number and location of isocenters.

**Results:**

Both weighted and unweighted versions of the k‐means algorithm were applied successfully to determine the number and position of isocenters. Comparing the two, both the within‐cluster sum of squares metric and the minimum target coverage metric resulting from the unweighted version were less than those from the weighted version. The average magnitudes of the differences were small (−0.2 cm^2^ and 0.1% for the within cluster sum of squares and minimum target coverage, respectively) but statistically significant (Wilcoxon signed‐rank test, *P* < 0.01).

**Conclusions:**

The differences between the versions of the k‐means clustering algorithm represented an advantage of the unweighted version for the within‐cluster sum of squares metric, and an advantage of the weighted version for the minimum target coverage metric. While additional treatment planning considerations have a large influence on the final treatment plan quality, both versions of the k‐means algorithm provide automatic, consistent, quantitative, and objective solutions to the tasks associated with SRS treatment planning using a single isocenter for multiple targets.

## 1 INTRODUCTION

Since its introduction over 60 yr ago, stereotactic radiosurgery (SRS) has been an effective method to treat both benign and malignant intracranial lesions.^1^, ^2^ Patients receiving SRS for the treatment of multiple brain metastases (mets) may be treated using one of several treatment machines or machine configurations. These include Gamma Knife (Elekta, Crawley, UK), CyberKnife (Accuray Inc., Sunnyvale, CA, USA), or a linear accelerator (linac) fit with a stereotactic cone or with a multileaf collimator (MLC). In the case of treatments using Gamma Knife, CyberKnife, or a linac with an SRS cone, multiple mets must be targeted individually and in sequence. However, linacs configured with MLCs provide an alternative. In this configuration, several targets may be included within a larger jaw‐defined field utilizing a single isocenter.^3^ Additional blocking is provided by the MLCs to achieve conformity around the targets. In this way, multiple brain mets can be irradiated simultaneously, improving the efficiency of treatment by reducing the amount of time the radiation beam is on and the amount of time the patient is on the table. Reducing the latter is also beneficial as the observed magnitude of intrafractional motion of some techniques has been shown to increase with the treatment duration.^4^, ^5^

This single isocenter for multiple targets technique, however, generates additional factors that must be considered, namely due to the unconventional position of the isocenter relative to the targets. For these treatments, the isocenter is typically not positioned in the center of the target, as would be the case if the target was being treated individually. Instead, it is positioned somewhere in the middle of the set of targets included within the field. With this unique configuration, three characteristic tasks emerge and must be addressed by the treatment planner. The first is the selection of the appropriate number of treatment plans and isocenters used to treat the full set of targets. The second is the identification of groups of targets to be treated simultaneously using the same isocenter. The third is the determination of the position of each isocenter. These tasks as presented here do not necessarily represent explicit steps in the treatment planning process, rather a conceptual framework of the novel elements characteristic to this planning technique.

The approach taken by the treatment planner in response to these tasks can have geometric and dosimetric consequences that are nontrivial due to the position of the off‐isocenter targets. One contributing factor is that the configuration and parameters of the linac are often described relative to the isocenter, and the machine's performance at the isocenter cannot necessarily be extrapolated to off‐isocenter positions. The isocenter also serves as the reference point for rotations described by the linac and many image guidance systems. A target not placed at the isocenter will be more sensitive to a rotation of a particular magnitude due to its radial distance from that point.^6^, ^7^, ^8^ Targets far from the isocenter may also be compromised by needing to be treated with the wider, peripheral MLC leaves rather than the narrower, central leaves. Furthermore, because of the MLC motion along a single direction, the ability of the MLCs to conform to the shape of each target is dependent on the collimator angle, beam angles, and the isocenter position.^9^ All of these factors can lead to detrimental dosimetric effects for off‐isocenter targets, necessitating consideration of the three emergent tasks.

Current practice tends to consider the first two of the three treatment planning tasks simultaneously. Clinicians are typically inclined to group the targets into as few treatment plans as possible in order to maximize the decrease in treatment time. However, this tends to increase the distance of each target to the isocenter, exacerbating their sensitivity to the linac's accuracy and precision as well as to any patient setup rotations. This also increases the likelihood that a target will exceed the range of the narrower, central MLC leaves. Furthermore, grouping the targets into as few plans as possible will tend to increase the jaw‐defined field size and the area where the radiation is blocked exclusively by the MLCs, increasing the contribution of leakage radiation. A reasonable approach to addressing these competing objectives is to group targets in close proximity into a single treatment field, while excluding faraway targets that might lead to a large increase in field size or might otherwise compromise the ability of the MLCs to conform to the targets. This strategy strives to balance the increased efficiency of the treatment and, at the same time, indirectly mitigate the deleterious effects of off‐isocenter targets and large jaw‐defined field sizes. In doing so, both the number of isocenters and the grouping of targets to be treated by each are decided.

After the first two tasks have been addressed, the third, the determination of the position of the isocenter, is often considered trivial. Each isocenter is simply placed near the center of the set of targets to be treated by that plan as a compromise that minimizes the distance between each target and the isocenter. This can be based on the center of target centroids^7^, ^10^ or the center of their combined volume.^6^, ^10^

These heuristic approaches seem reasonable and effective. However, they remain subjective with no guarantee of consistency patient‐to‐patient or treatment planner‐to‐treatment planner. Considering the increasing prevalence of the single isocenter for multiple targets technique and the potential dosimetric effects of inadequately addressing the technique‐specific considerations, an objective, more robust, and automatic approach to the three emergent tasks is necessary.

Fortunately, the simple and robust k‐means clustering algorithm naturally lends itself to providing a more consistent solution. A common, unsupervised learning algorithm, the k‐means clustering algorithm was described by MacQueen in 1967.^11^ It clusters data by minimizing the within‐cluster variance or within‐cluster sum of squares, equivalent to the sum of squared Euclidean distance between each data point and the cluster centroid. A predetermined number of classes is provided as input to the algorithm along with the observed dataset. The results of the algorithm are the optimal values of mean reference points and the classification of each data point to one of these reference points. The appropriate number of classes can be inferred from repeating the algorithm with varying numbers of classes and considering the change in the within‐cluster variance. In the context of SRS treatment planning using a single isocenter for multiple targets, the number of classes is analogous to the number of treatment plans and isocenters used to treat the full set of targets. The results of the algorithm provide groupings of targets and the locations of isocenters that minimize the sum of squared distance between each target and the isocenter. In addition, it provides a quantitative metric (i.e., the within‐cluster sum of squares) that can be used to evaluate treatment approaches of various numbers of isocenters. The application and advantages of this algorithm to multitarget SRS treatment planning have yet to be described in the literature.

The purpose of this work was to present the application of the k‐means clustering algorithm to SRS treatment plans featuring a single isocenter for multiple targets. The algorithm is a tool that promotes an objective and consistent treatment planning approach while automatically addressing three emergent tasks characteristic of this treatment technique.

## 2 METHODS

### 2.A Data acquisition

To demonstrate the application of the k‐means clustering algorithm to SRS treatment planning, we retrospectively selected 30 patients that had been recently treated using the single isocenter for multiple targets technique (Varian TrueBeam 2.0, Varian Medical Systems, Palo Alto, CA, USA). Patient selection was otherwise random. For each met, the 3D coordinates of the geometric centroid and the radius of the sphere with equivalent volume were determined from the treatment planning system (Eclipse v13.6, Varian Medical Systems, Palo Alto, CA, USA). These data served as the input to the implementation of the k‐means algorithm described below.

### 2.B Using the k‐means clustering algorithm to group targets and select isocenter positions

For this work, the patient‐specific sets of met centroids and radii were fed into a software interface developed in‐house that featured the Matlab implementation of the k‐means clustering algorithm (*kmeans*, Mathworks, Natick, MA, USA). The software varied the number of classes used by the algorithm between one and *m*, the patient‐specific number of mets. For each instance, the algorithm determined the optimal classification of the *m* mets into groups to be treated by *k* isocenters, as well as the position of the isocenters that minimized the within‐cluster variance. The within‐cluster variance is equivalent to the sum of squared Euclidean distance between each met and its isocenter (i.e., within cluster sum of squares). The algorithm repeated this process 100× to avoid local optimal classification results.

In addition to this conventional application of the k‐means algorithm, we also implemented a weighted version of the algorithm. For this version, the data point representing each met's geometric centroid was replicated so that the number of data points was inversely proportional to the size (radius) of the met relative to the others. This directed the algorithm to “favor” smaller mets which are more sensitive to rotations by positioning the resulting isocenter closer to them. As an additional result, any met that is far enough away from the isocenter to be beyond the range of the narrower, central leaves, will more likely be of a larger size where the use of the wider, peripheral leaves is of less consequence.

The within‐cluster sum of squares resulting from the weighted and unweighted versions of the algorithm were compared using a Wilcoxon signed‐rank test as a paired, nonparametric statistical test. The instances where the number of classes was equal to the number of mets were excluded because both versions of the algorithm would yield the trivial solution where an isocenter was placed in the center of each met and the within‐cluster sum of squares is zero.

### 2.C Evaluating the target coverage resulting from the k‐means clustering algorithm

To better estimate the dosimetric effects of the algorithm's results, we converted the results from both the weighted and unweighted versions of the algorithm into a target coverage metric derived from that described previously.^8^ In the original description of the metric, a sphere of volume equivalent to the target in consideration is displaced by a geometric transformation relative to an origin representing the isocenter. The coverage metric value is the relative volume of overlap between the displaced sphere and another, stationary sphere that represented a particular isodose line. In this work, we considered the relative volume of overlap between a displaced sphere and a stationary sphere that both represented the volume of the target in consideration. Furthermore, we focused on transformations comprised of a single rotation only. Because the rotations can occur in any anatomic plane, the rotation for this metric was considered to occur around an axis that was perpendicular to a line between the target centroid and the isocenter. This maximized the displacement of the target, and therefore represented a worst‐case scenario regarding target coverage. Under these circumstances, calculation of the coverage metric was simplified as represented in eq. (1). In eq. (1), R is the distance from the center of the target to the isocenter, θ is the angle of rotation in degrees, and r is the radius of the sphere of equivalent volume.

(1) $$targetCoverage\left(\%\right)=100*\left[1-\frac{3}{2}\left(\frac{\pi R\theta }{360r}\right)\;+\;\frac{1}{2}{\left(\frac{\pi R\theta }{360r}\right)}^{3}\right]$$

In this work, we specifically considered rotations of 0.5° as an example transformation. A rotation of this magnitude is typical of those observed clinically. Because the algorithm considered each target and each class simultaneously, we represented the solution by considering the minimally covered target.

As was the case with the within‐cluster sum of squares described above, the results of the weighted and unweighted versions of the algorithm were compared in terms of minimum target coverage using a Wilcoxon signed‐rank test.

### 2.D Applying the k‐means clustering algorithm prospectively to an example patient

Lastly, to demonstrate how the k‐means algorithm is used prospectively on a patient‐specific basis, the results of the unweighted version of the algorithm are presented with both the within‐cluster sum of squares and the minimum target coverage as functions of the number of isocenters for a representative example patient.

## 3 RESULTS

The 30 patients selected for this work represented a total of 168 mets with a mean of 5.6 mets per patient (range: 2–17 mets/patient). The radii of the spheres with volumes equivalent to the mets ranged from 0.3 to 1.2 cm with a mean of 0.5 cm.

Table 1 provides the comparison of the results from the weighted and unweighted versions of the k‐means algorithm. The within‐cluster sum of squares resulting from the unweighted version of the algorithm was, on average, less than that from the weighted version. While small in magnitude, the difference was nonetheless statistically significant according to a Wilcoxon signed‐rank test. The minimum target coverage resulting from the weighted version of the algorithm was greater than that from the unweighted version by a small magnitude that was also statistically significant.

**Table 1:** Results of the statistical comparison of the weighted and unweighted versions of the k‐means algorithm for the within‐cluster sum of squares metric (WCSS) and the minimum target coverage metric (minTargetCov)

Metric comparison	Median difference (Range)
$WCSS_{unweighted}-WCSS_{weighted}$	−0.2 cm^2^ (−5.6 cm^2^ to 0.7 cm^2^)^a^
$minTargetCov_{weighted}-minTargetCov_{unweighted}$	0.1% (−0.5% to 2.1%)^a^

a Statistically significant (*P* < 0.01).

**Figure 1:**
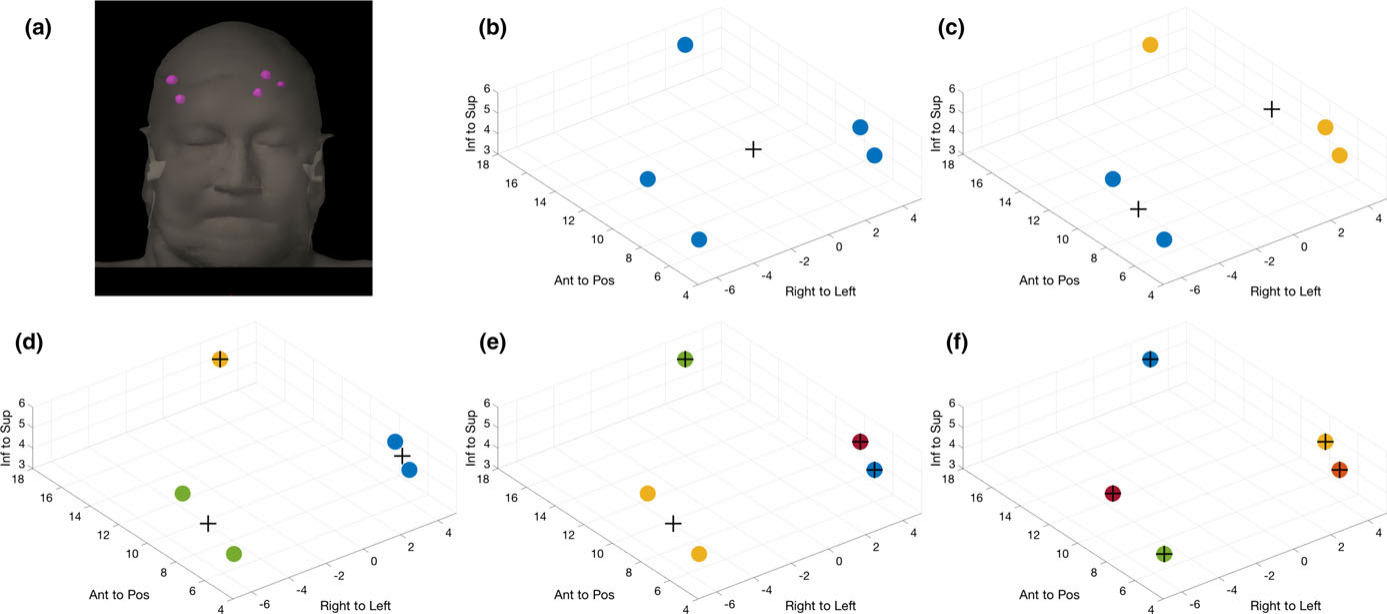
Grouping of the example patient's five mets. (a) The anatomic distribution of the five mets. (b) The grouping of the mets resulting from the k‐means algorithm when treated with one isocenter, (c) two isocenters, (d) three isocenters, (e) four isocenters, and (f) five isocenters. Targets are depicted as spheres that are color‐coded by group, and the isocenters are depicted as (+). All anatomic coordinates are in units of centimeters.

The prospective analyses for the example patient are depicted in Figs. 1 through 3. This patient was treated for five mets, each with either a 0.4 cm or 0.5 cm equivalent sphere radius. Figure 1 represents the grouping of the mets for the scenarios where the five mets are to be treated with one to five isocenters.

**Figure 2:**
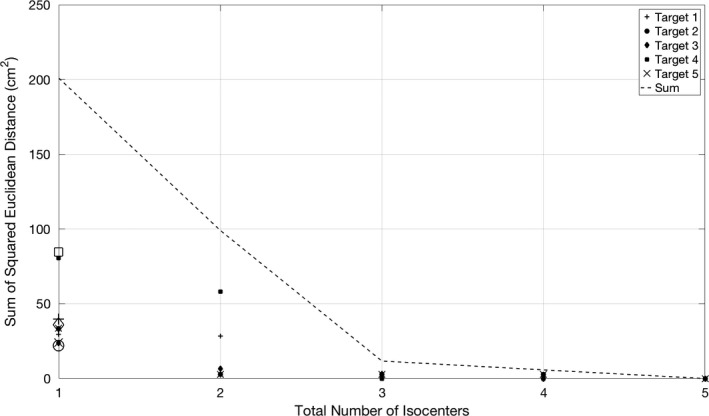
The squared Euclidean distances between each target and its isocenter and the within‐cluster sum of squares. These values are the results of the unweighted version of the k‐means algorithm for the example patient as a function of the number of isocenters. An “elbow” in the curve is observed with three isocenters. The open markers at *k* = 1 denote the values resulting from the isocenter used for the patient's actual treatment. The within‐cluster sum of squares is represented by the dotted line.

In Fig. 2, the within‐cluster sum of squares from the unweighted version of the algorithm is plotted as a function of the number of isocenters. Also plotted are the squared Euclidean distances from each target to its isocenter. These are the components that make up the within‐cluster sum of squares metric. It is apparent that the value of this metric decreases with the addition of each isocenter; however, the effect is of diminishing returns as successive isocenters are added. The “elbow” at *k* = 3 represents the point where a user may decide that the cost of adding subsequent isocenters is no longer worth the decreasing benefit of minimizing the distance to the isocenters. However, there are other clinical factors that must be considered in making this decision as presented in the Discussion.

**Figure 3:**
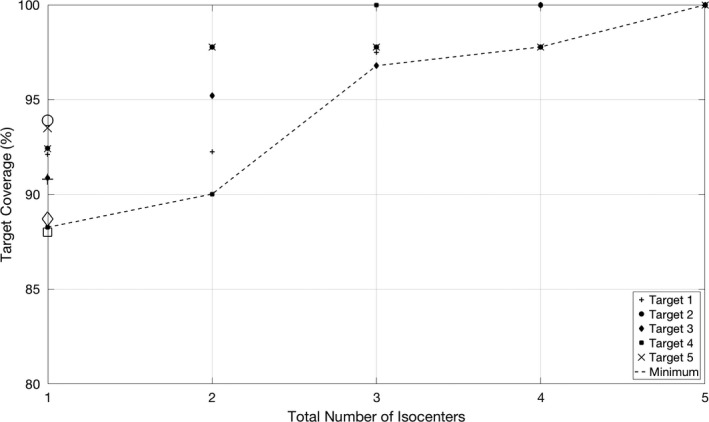
Target coverage metric for each met and the minimum target coverage resulting from the unweighted version of the k‐means algorithm after a 0.5° rotation for the example patient as a function of the number of isocenters. The open markers at *k* = 1 denote the values resulting from the isocenter used for the patient's actual treatment.

Figure 3 presents the target coverage metric for each met along with the minimum target coverage value from the unweighted version of the algorithm plotted as a function of the number of isocenters. The coverage is improved with the addition of isocenters; however, like Fig. 2, the effect is of diminishing returns. An “elbow” is also present in the curve at *k* = 3.

## 4 DISCUSSION

This work demonstrates the application of the k‐means clustering algorithm as a practical tool for addressing the emergent considerations characteristic of SRS treatment planning using a single isocenter for multiple targets. The algorithm can automatically group the targets to be treated with an individual isocenter as well as position the isocenter in a consistent and objective manner. A weighted version of the algorithm can be used to “favor” smaller targets that are more sensitive to rotations. This is a direct result of the mathematics behind the k‐means clustering algorithm. Because the weighted version replicates data points to a degree inversely related to their relative size, the isocenter that results will necessarily move toward these smaller targets to minimize the sum of squared distances, which will in turn increase the value of their target coverage metric. Lastly, quantitative metrics from the algorithm can be used to evaluate the effect of the number of isocenters.

Previously, we described the use of a coverage metric to consider the effects of translations and rotations when determining patient‐specific SRS action limits.^8^ Besides that work, the most closely related analysis of the rotational sensitivity of this technique is that conducted by Roper et al.^7^ In it, the authors simulated rotations around all three orthogonal anatomic axes. They then generated multivariate regression models relating rotational errors to target size, position, and coverage. Our approach is different in that it identifies the patient‐specific sensitivity to rotations without considering any population‐based statistics. We believe a patient‐specific approach is preferred as the results will depend on the details of the anatomy, disease, and treatment goals. For this reason, we have refrained from making generalized recommendations regarding a maximum allowed distance between a target and its isocenter. Instead, we have focused on providing clinicians with a technique that can be employed on a patient‐by‐patient basis. To that end, we implicitly assumed that the effect of rotations on target coverage is reflected in the relative overlap of a spherical target before and after a rotation. The models of Roper et al. have the advantage of having considered the actual target contour in relation to the actual dose distribution to determine the dosimetric effect of a rotation. While our approach is limited to a geometric approximation, we believe it suffices nonetheless. Brain mets tend to be spherical in shape, and so using a spherical approximation of the target is not a large departure from reality. We were also able to exploit this spherical geometry to represent the composite transformations around three orthogonal axes as a single rotation around another appropriately oriented axis. In our target coverage metric, we focused on the worst‐case scenario when evaluating the sensitivity of each met to rotations. This recognizes that rotations are not limited to the conventional anatomic dimensions, even if that is how they are represented by image guidance systems. In addition, our application of the metric is a relative one for the sake of assessing the comparative value of different target groupings and isocenter positions. It is not intended to predict the absolute difference in any particular dosimetric parameter. Lastly, the residual discrepancy between this coverage metric and the actual dosimetric effects of rotations are likely overshadowed by additional treatment planning considerations.

While the k‐means algorithm is an effective way to address the emergent considerations regarding the technique of using a single isocenter for multiple targets, it does not address all of the factors that influence the sensitivity of a treatment plan to rotations. The geometric distance of a target to the isocenter is only one of several factors that influences how optimal the position of the isocenter is from a clinical perspective. Additional factors include other characteristics about the target (e.g., its shape), as well as details of the dose distribution stemming from the treatment plan and machine configuration (e.g., dose gradient, prescription, normalization, number of beams or arcs, gantry angles, couch angles, and collimator angles). In the treatment planning process, however, the isocenter is typically determined before many of these other factors are decided. Short of addressing all these factors simultaneously in order to determine the truly optimal isocenter position, selecting the isocenter according to the geometric factors available at the beginning of the treatment planning process is sensible.

Other authors have described placing the isocenter in the middle of the target centroids^7^ or in the middle of a structure representing the Boolean union of all targets.^6^ The former leads to the same result as the unweighted version of our k‐means algorithm; the latter yields the opposite effect of the weighted version by drawing the isocenter toward the larger targets which is undesirable for the sake of minimizing sensitivity to rotations.^6^, ^7^, ^9^, ^10^ Our k‐means algorithm approach, therefore, can place the isocenter in a manner comparable to a common technique (using the unweighted version) or with the recommended characteristic of being closer to small targets to a degree inversely proportional to their size (using the weighted version).

Our observed differences between the weighted and unweighted versions of the algorithm were small but statistically significant. The unweighted version was superior regarding the within‐cluster sum of squares while the weighted version was superior when considering the minimum target coverage. That the results were ultimately very similar between the two versions is in part due to the relatively similar sizes of most of the mets observed in the patients studied. Were a patient to exhibit targets of considerably different sizes that were to be treated using a single isocenter, the weighted version of the algorithm may be more appropriate in order to ensure the smaller targets maintain sufficient coverage. This is consistent with the observations and recommendations of previous authors.^7^, ^10^ If the targets are of similar sizes, either version of the algorithm is likely sufficient as the discrepancy is small, particularly when considering the effects of the other subsequent components of the treatment planning process as described above.

Kang and McNutt comment on the choice of grouping targets into different treatment plans and the effect that it can have on the resulting dosimetry.^9^ Their approach was to exhaustively search through each combination of the six lesions of an example patient in order to identify the most advantageous grouping. While this approach is complete, with an increasing number of targets the task soon becomes prohibited by the combinatorial explosion of the number of possible groupings. Our k‐means algorithm approach efficiently identifies the optimal grouping, at least in terms of minimizing the within cluster sum of squares. It is interesting to note that the work of Kang and McNutt is focused on optimizing the collimator and couch angle to avoid suboptimal MLC configurations. In doing so, they observed the grouping of targets according to geometric proximity alone was not optimal when considering the orientation and motion of the MLCs. This illustrates the discussion above regarding the additional factors that influence the final quality of the treatment plan.

When using the k‐means approach to compare treatments with various numbers of isocenters, proper clinical context is necessary. Adding an isocenter will certainly decrease the distance to the targets, and may lead to meaningful improvements regarding the sensitivity of each target to rotations. However, there are costs incurred that are not reflected in the distance alone. These include the increase in treatment time, which in turn may compromise patient comfort and localization. Morrison et al. manually added a second and third isocenter to SRS patients previously treated with a single isocenter.^12^ They observed that while the additional isocenters reduced the distance from each target to the isocenter, the changes in several dosimetric parameters were not large, leading them to conclude that a single isocenter is sufficient. Our analysis suggests that there could be considerable gains from adding a second or third isocenter, and our approach provides a quantitative way to compare these options, albeit not via clinical dosimetric parameters. Therefore, clinicians must recognize that the additional treatment planning and delivery considerations are not reflected in the figures presenting distance or coverage metrics, and must keep these factors in mind when evaluating the results of an approach like ours.

Despite these limitations, our implementation of the k‐means clustering algorithm to group targets and position the isocenter has numerous advantages. It is a natural application of a simple and robust algorithm that is fast and objective. It generalizes to the patient‐specific number and configuration of targets and requires minimal effort on behalf of the treatment planner. It simultaneously optimizes the grouping of targets to be treated concurrently, selects the position of each isocenter, and elucidates the impact of the number of isocenters. As a result, our approach provides clinicians with a simple yet effective tool to mitigate the deleterious effects of rotations and excessive field sizes, thereby improving intracranial SRS treatment plans using a single isocenter for multiple targets.

## 5 CONCLUSION

Weighted and unweighted versions of the k‐means clustering algorithm can be used to address the additional SRS treatment planning tasks when using a single isocenter for multiple targets. While factors such as the final dose distribution also heavily influence the sensitivity of off‐isocenter targets to rotations, the k‐means clustering algorithm provides an approach to the initial geometric considerations of the planning process in a way that is automatic, consistent, quantitative, and objective.

## CONFLICTS OF INTEREST

The authors declare no conflict of interest.
